# Gynecomastia: physiopathology, evaluation and treatment

**DOI:** 10.1590/S1516-31802012000300009

**Published:** 2012-07-12

**Authors:** Alfredo Carlos Simões Dornellas de Barros, Marcelo de Castro Moura Sampaio

**Affiliations:** I MD, PhD. Coordinator of the Mastology Department, Hospital Sírio-Libanês, and Researcher in the Discipline of Human Structural Topography (LIM 02), Faculdade de Medicina da Universidade de São Paulo (FMUSP), São Paulo, Brasil.; II MD, MSc. Plastic Surgeon in the Mastology Department, Hospital Sírio-Libanês, São Paulo, Brasil.

**Keywords:** Breast, Gynecomastia, Surgery, Adolescent, Endocrine system diseases, Mama, Ginecomastia, Cirurgia, Adolescente, Doenças do sistema endócrino

## Abstract

Gynecomastia (GM) is characterized by enlargement of the male breast, caused by glandular proliferation and fat deposition. GM is common and occurs in adolescents, adults and in old age. The aim of this review is to discuss the pathophysiology, etiology, evaluation and therapy of GM. A hormonal imbalance between estrogens and androgens is the key hallmark of GM generation. The etiology of GM is attributable to physiological factors, endocrine tumors or dysfunctions, non-endocrine diseases, drug use or idiopathic causes. Clinical evaluation must address diagnostic confirmation, search for an etiological factor and classify GM into severity grades to guide the treatment. A proposal for tailored therapy is presented. Weight loss, reassurance, pharmacotherapy with tamoxifen and surgical correction are the therapeutic options. For long-standing GM, the best results are generally achieved through surgery, combining liposuction and mammary adenectomy.

## RESUMO

A ginecomastia (GM) é caracterizada pelo aumento do volume mamário em homens, provocada por proliferação glandular e depósito de gordura. É comum e pode ocorrer em adolescentes, adultos e idosos. O objetivo desta revisão é discutir a fisiopatologia, etiologia, avaliação clínica e terapia da GM. Um desequilíbrio entre estrogênios e androgênios é reconhecido como a condição fundamental para a determinação do quadro, podendo ser atribuído a fatores fisiológicos, tumores ou distúrbios endócrinos, doenças não-endócrinas, uso de drogas ou ser de causa idiopática. A avaliação clínica deve enfocar a confirmação diagnóstica, a procura de uma causa específica e a classificação em graus de gravidade para orientar o tratamento. Um roteiro de conduta individualizada é apresentado, variando entre perda de peso e explicações verbais, tratamento com tamoxifeno e correção cirúrgica. Para casos de GM persistente, os melhores resultados são obtidos em geral mediante abordagem cirúrgica, combinando lipoaspiração e adenectomia mamária.

## INTRODUCTION

Gynecomastia (GM) is a benign condition characterized by enlargement of the male breast, which is attributable to proliferation of the glandular tissue and local fat deposition. It can be physically uncomfortable, psychologically distressing and may have a negative impact on self-confidence and body image. Pseudogynecomastia is common in obese men, and consists of lipomastia alone, without glandular proliferation. This condition, together with neonatal transient breast hypertrophy caused by the high estrogenic milieu of pregnancy, should not be considered to be true GM, and these are beyond the scope of this paper.

Male breast tissue proliferation can occur at all ages and may be unilateral or bilateral. It has been estimated that 30% to 60% of boys exhibit GM during adolescence and that at least one third of the adult male population may be affected. The differences between samples can be accounted for by the criteria used to define them and the diversity of the samples studied.^1^^,^^2^^,^^3^ The highest prevalence of GM is found in old age, when it is detected in up to 65% of men.^4^^,^^5^

GM is common and clinically important. Nevertheless, major gaps in knowledge regarding its modern epidemiology exist, and it has not been proven whether the apparent rise in its incidence is true. Nonetheless, over recent decades, there have been substantial increases in the use of anabolic steroids and in environmental contamination with xenoestrogens or estrogen-like substances that, at least theoretically, can stimulate glandular proliferation of the male breast.^6^^,^^7^

This review addresses the pathophysiology, etiology and clinical evaluation of GM and discusses the selection of patients for tailored therapy, which remains a challenge for physicians. We performed a search for published scientific papers in PubMed, SciELO and the Cochrane Database of Systematic Reviews (Table 1), covering 2000 to 2011.

**Table 1.** Database search results

Database	Search	Results
PubMed	gynecomastia	13 reviews
	gynaecomastia	3 clinical trials
		1 case-control study
SciELO	ginecomastia	5 case series
	gynecomastia	2 reviews
Cochrane Library	gynecomastia	none
	gynaecomastia	

## PHYSIOPATHOLOGY

It is well known that male breast tissue contain protein receptors for estrogens and androgens.^6^^,^^8^^,^^9^ Whereas estrogens stimulate the proliferation of the mammary ductules, androgens, on the contrary, inhibit the process.^10^^,^^11^ A hormonal imbalance between these factors is the key hallmark for GM generation.^12^^,^^13^^,^^14^ The estrogen/androgen imbalance may be attributable to increased levels of free estrogens secreted by the testes or adrenal glands, extraglandular aromatization of estrogen precursors, decreased estrogen degradation, exposure to estrogen-like chemicals or exogenous estrogens and use of drugs that cause displacement of more estrogen than androgen from sex hormone-binding globulin (SHBG).^10^^,^^13^ On the other side, the imbalance may result from decreased androgen production in the testes, increased binding of androgens (relative to estrogens) by SHBG, altered androgen metabolism, drug-induced displacement of androgens from their receptors and androgen receptor defects.^15^^,^^16^^,^^17^

In males, the testes secrete about 95% of the circulating testosterone, 15% of the estradiol and 5% of the estrone that are produced daily. In normal men, the serum concentration of estrogens is very low. Most of the estrogens (80%) are produced by peripheral conversion of two precursors, androstenedione and testosterone, respectively in estrone and estradiol, under the influence of the enzyme aromatase, which plays a pivotal role in male secretion of estrogens. Peripheral conversion occurs mainly in the intramammary and subcutaneous fat, but is also seen in the liver, skin, muscles and kidneys. Aromatase activity increases both with age and with elevation of the body mass index.^13^^,^^14^^,^^15^^,^^16^

Continuous environmental exposure to substances with weak estrogen-agonist action, probably due to epigenetic mechanisms, theoretically can induce the development of GM. Endocrine-disrupting chemicals in consumer products, air pollutants, radiation, organochlorine pesticides, plastics, plasticizers, fuels and polycyclic aromatic hydrocarbons are listed among these substances. Improved exposure assessment methods will help advance future epidemiological and related interdisciplinary research on the relationship between the environment and GM.^18^

The first study showing the link between GM and environmental chemicals was published by Durmaz et al.^19^ These authors compared the plasma phthalate levels in 40 diagnosed pubertal GM cases with the levels in 21 age-matched boys without GM. They observed that phthalate metabolite concentrations in the plasma were significantly higher in the adolescents with GM than in the control group (P < 0.01). Phthalates are esters of alpha-phthalic acid with antiandrogenic and estrogenic effects that are found in personal care products (cosmetics, perfumes and clothing), paints, solvents, insecticides, plasticizers, food, water and pharmaceuticals.^6^^,^^20^^,^^21^

Reinforcing the evidence suggesting that there is a relationship between chemicals and GM, it is worthwhile mentioning the epidemic onset observed among Haitian refugees in 1981 about four months after arrival in United States detention centers.^22^ After analyzing all identifiable environmental exposures, it was then found that phenothrin, a multi-insecticide contained in sprays that they had used was the causative agent.^23^ It is now widely known that phenothrin has antiandrogenic activity.

There is evidence that enlarged male breast glands contain luteinizing hormone (LH) and human chorionic gonadotropin (hCG) receptors.^24^ The role of these receptors has yet to be elucidated, but it is reasonable to suppose that their activation could decrease the androgenic effects by altering the local metabolism. It has also been reported that LH and hCG reduce the concentration of androgen receptors in the skin.^25^

Findings of lobular structures in microscopic analysis on GM tissue are very rare. Progesterone appears to be required to form true glandular acini acting in synergy with insulin growth factor-1 (IGF-1).^26^ Under some clinical conditions, such as hyperthyroidism and hepatic cirrhosis associated with GM, it is possible to detect elevated serum progesterone concentrations.^27^^,^^28^

Another hormonal action that stimulates breast tissue in men is observed secondary to hyperprolactinemia. Prolactin receptors have been demonstrated in GM, and hyperprolactinemia probably plays an indirect role in GM, since it causes central hypogonadism and alters the androgen/estrogen ratio.^14^^,^^29^^,^^30^ However, it is clear that most men with GM do not have elevated serum prolactin levels and not all men with hyperprolactinemia develop GM.^14^ Nonetheless, it has been shown in cultured breast cancer cells that prolactin and progesterone receptors may be coexpressed and may cross-regulate each expression.^31^ Similar crosstalk may exist in relation to growth hormones, thus giving rise to a permissive role for estrogen activity, probably via the IGF-1 receptor.^32^

GM is a multifactorial disease and many conditions may be associated with it. Table 2 outlines the various specific causes of GM. According to Braunstein,^33^ almost two-thirds of the patients have physiological GM (approximately 25%), no underlying detected abnormality (idiopathic, approximately 25%) or drug-induced breast development (up to 20%). The frequencies of some of the remaining causes have been estimated as follows: cirrhosis, 8%; primary hypogonadism, 8%; testicular tumors, 3%; secondary hypogonadism, 2%; hyperthyroidism, 1.5%; and renal disease, 1%.

**Table 2.** Etiology of gynecomastia

Causes	
Physiological factors	Puberty or aging
Endocrine tumors	Testicular, adrenocortical or pituitary tumors, or ectopic hCG-secretion
Endocrine dysfunctions	Hypogonadism, hyperthyroidism, obesity or refeeding
Non-endocrine diseases	Cirrhosis, renal failure or HIV
Drug-induced factors	Medications, anabolic steroids or illicit drugs
Idiopathic factors	

### Pubertal GM

Mild degrees of pubertal GM generally appear at 13 or 14 years of age, last for 6-12 months and then spontaneously regress in 95% of the cases.^13^ The glandular enlargement may be asymmetric and tender. The hypertrophy may reach severe proportions, leading to an effeminate appearance in boys. Such occurrences may alter self-perceptions, especially in the sexual sphere.^34^

Relative excess of serum levels of estrogens compared with androgens is implicated in the pathogenesis, due to estradiol production rising sooner than testosterone production. Some other factors may also interact, and usually there is concomitant elevation in serum IGF-1 concentrations. Interestingly, a family history has been elicited in more than half of the patients with persistent pubertal GM.^17^^,^^34^

### Aging GM

Older men over the age of 65 years often present relative hypogonadism with a decline in plasma testosterone levels, elevation of SHBG and decrease in free testosterone. Furthermore, there is progressive adiposity favoring peripheral aromatase activity.^35^^,^^36^ Several comorbidities may be common at this age, and any medication used may contribute towards provoking or aggravating GM.^14^

### Endocrine tumors

Benign testicular tumors (Sertoli or Leydig cell tumors) may secrete estradiol. Secondary suppression of LH levels interferes negatively with testosterone synthesis. The elevated estrogen levels raise the serum concentration of SHBG, which preferentially binds testosterone, thereby lowering the free testosterone levels.^12^

Choriocarcinoma and other germ cell tumors produce hCG and stimulate testicular cells (Leydig) to secrete estradiol, and furthermore, often cause GM. These tumors may be palpable or be detected only by means of ultrasound. Other hCG secreting tumors of ectopic origin may lead to GM (e.g. carcinomas of the lungs, liver, stomach and kidneys).^13^^,^^14^^,^^15^^,^^17^

Pituitary adenomas producing prolactin (prolactinomas) may also induce GM.^17^

Adrenocortical tumors are generally large malignant types of neoplasia, with predominant incidence in young and middle-aged men. They are feminizing tumors with direct secretion of estrogens and steroid precursors, like androstenedione. The serum estrogen elevation also suppresses LH-mediated testosterone production.^14^

### Endocrine dysfunctions

Severe hyperthyroidism increases serum SHBG. Since estradiol binds less avidly to SHBG than does testosterone, there appears to be an increase in the ratio of free estradiol to free testosterone, thus resulting in clinically evident GM in 10 to 40% of the patients.^37^

Primary gonadal failure as a result of testicular trauma, chemotherapy, mumps, orchitis and leprosy can cause GM by lowering the serum testosterone levels, inducing elevation of LH and stimulating the remaining Leydig cells to secrete estrogens.^12^ Klinefelter syndrome is a chromosomal disorder (47 XXY karyotype) associated with hypogonadism and infertility; in these men, GM is seen in almost 70%. The reason why the presence of an extra X chromosome is linked to GM is unclear.^14^^,^^38^ Male pseudohermaphroditism with Morris syndrome (testicular feminization) is often associated with normal female breast appearance due to gonadal estrogen production.^33^ Low testosterone and elevated LH levels indicate primary hypogonadism. Findings of low testosterone levels with normal LH assays denote secondary testicular failure.^33^

It is noteworthy that men with long-standing type 1 diabetes may develop diabetic mastopathy, presenting hard diffuse enlargements of one or both breasts.^39^ An inflammatory lesion characterized by lymphocytic infiltration of the mammary ducts and lobules is found microscopically.^39^

Other endocrine-metabolic conditions related to the development of GM include metabolic syndrome, refeeding after severe starvation and substantial weight loss and functional hyperprolactinemia.^12^^,^^14^

### Non-endocrine diseases

The chief sex hormone abnormalities in cases of liver cirrhosis are decreased serum testosterone levels and increased estradiol levels.^39^ The mechanisms that cause liver cirrhosis to lead to GM in a small percentage of cases have yet to be elucidated. The causes are probably multifactorial. Men with chronic renal failure are often hypogonadal, with defects in testicular steroidogenesis.^12^ Many of these men develop GM.

In men with HIV, GM occurs in 2-3%.^40^ GM can be triggered either by lipodystrophy or by highly active antiretroviral therapy.

### Drug induction

Drug-induced GM merits deep consideration as it may account for as many as 25% of all cases of new-onset GM in adults. Even though the mechanisms through which a long list of drugs can cause GM are not fully clear, they are derived from estrogen-like activities, stimulation of testicular production of estrogens, inhibition of testosterone synthesis or blockade of androgen action.^12^^,^^41^

One of the medications most frequently associated with GM is the diuretic spironolactone, which is a competitive antagonist of aldosterone. Spironolactone also inhibits testosterone production in the testes, enhances the aromatization of testosterone to estradiol and binds to androgen receptors in some tissues, thereby acting as an antiandrogenic substance.^41^^,^^42^

Androgen deprivation therapy for prostate cancer frequently presents GM as a side effect. The incidence of GM depends on the type and duration of the hormone therapy, but it can be as high as 40-70%.^43^

Some illicit drugs, such as cocaine, heroin and amphetamines, and other abused drugs, are commonly associated with GM. Marijuana is believed to interfere with estrogen receptors and acts as a phytoestrogen.^44^

Doping with anabolic androgenic steroids, gonadotropins and growth hormones for power sports and weight training is rampant. In male athletes, these iatrogenic drugs may suppress spermatogenesis and/or induce GM.^45^

Table 3 outlines the common pharmaceutical products that may cause GM with prolonged use.

**Table 3.** Common medications causing gynecomastia^41^

Type of agent	Medications
Antiandrogens	Bicalutamide, cyproterone, flutamide, finasteride, spironolactone
Antibiotics	Isoniazid, ketoconazole, metronidazole
Antihypertensives	Amlodipine, captopril, enalapril, nifedipine, reserpine, verapamil
Chemotherapeutic agents	Cyclophosphamide, methotrexate
Diuretic	Spironolactone
Gastrointestinal agents	Cimetidine, omeprazole, metoclopramide, ranitidine
Hormones	Androgens, anabolic steroids, estrogens, growth hormone
Psychiatric agents	Diazepam, haloperidol, phenothiazine, tricyclic antidepressants
Others	Amiodarone, antiretrovirals, digitalis, domperidone, statins, theophylline

### Idiopathic GM

So far, at least 20 clinical conditions and 30 medications have been implicated in relation to causing GM. However, the etiology of GM is still only understood to a limited extent, and up to 50% of the cases may have no obvious cause.^33^ Given the high frequency of idiopathic GM, we hypothesize that multiple environmental endocrine disruptors are likely to be involved in excessive breast development in men.

## EVALUATION

Clinical evaluations on men with enlarged breast tissue have three phases: 1) making the diagnosis of true GM; 2) trying to find an etiological factor to further guide case management; and 3) classifying into severity grades.

### 1. Making the diagnosis

Differentiation of true GM from pseudogynecomastia (local fat deposition alone) and tumors is based on physical examination by means of inspection and palpation. Initially, the patient must disrobe from the waist upwards, for inspection in a seated position: with the arms relaxed, with the arms raised, and with the hands pressed against the hips to contract the pectoral muscles. After palpation of the axillary nodes, the patient is asked to lie down in a supine position with his hands clasped beneath his head. First, the examiner carefully flat-palpates with his fingers to detect glandular tissue, and then, with his thumb and forefinger separated, slowly brings the fingers together from either side of the breast.^13^ In true GM cases, a disc of firm tissue, concentric with the nipple-areolar complex, is felt. Patients with pseudogynecomastia do not show mound resistance of this nature, and no firm tissue is found.^13^

Breast carcinoma usually consists of a unilateral hard irregular mass, located outside the areola and may be accompanied by skin dimpling, nipple retraction and axillary lymphadenopathy. Other local problems, such as dermoid cysts, lymphangiomas, lipomas, post-trauma hematomas, neurofibromas and several benign tumors are usually easily distinguished. Any lesion that is suspected to be malignant should be evaluated by means of core-needle or excisional biopsies.

GM is clinically bilateral in approximately half of the patients.^13^ Nipple discharge is very uncommon.^13^^,^^14^^,^^15^^,^^16^^,^^17^

Ruling out the possible presence of breast carcinoma is paramount, particularly in adults with unilateral enlargements, family histories of breast cancer or Klinefelter syndrome. Mammography is the primary imaging method when there is any suspicion of cancer. As a predictor of malignancy, mammography has sensitivity of 90% and specificity of 92%.^46^

Sonography has been widely used in GM cases. It is important to recognize the various patterns of GM in order to avoid unnecessary worries. The typical findings include hypoechoic retroareolar masses (nodular, poorly defined or flame-shaped), with increased anteroposterior depth at the nipple.^47^

**Figure 1.**
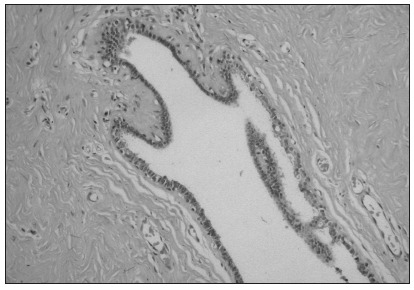
Microscopic findings of gynecomastia characterized by dense fibrous stroma and dilated ductules without lobules (hematoxylin and eosin, 200 x).

If differentiation between GM and breast carcinoma cannot be made on the basis of clinical and imaging findings, the patient should undergo percutaneous biopsy. Microscopically, GM is characterized by proliferation of the ductules, without terminal acini, in fibroconnective stroma (Figure 1). In the early onset stage (florid phase), there is extensive ductal hyperplasia; over time, the glandular elements become less prominent and fibrosis becomes the main finding (fibrous phase). Although it might be tempting to try to establish a correlation between etiological factors and histological appearance, no such correlation has ever been identified.

The current wisdom is to endorse the concept that men with GM are not at high risk of breast cancer.^48^ However, certain factors linked with increased incidence of GM also are related to cancer, such as estrogen use and androgen deficiency.^49^^,^^50^ A meta-analysis on seven case-control studies revealed that the breast cancer risk is slightly higher in males with GM.^51^ Klinefelter syndrome is the only condition in which the risk of cancer is worrisome, with a 50-fold higher risk of developing breast cancer than among men in the general population.^52^

### 2. Trying to find an etiological factor

A detailed history, with attention given to age, medications, duration and onset of breast enlargement, symptoms of tenderness or pain, recreational drug use and anabolic steroid use, is crucial. The general physical examination may reveal signs of thyroid disease, hypogonadism or other conditions. Abdominal masses may be found in patients with adrenocortical carcinomas. The investigation should specifically pay attention both to the breasts and to the genitals.

Further work-up includes screening laboratory tests and ultrasonography on the adrenal glands and testes. However, in practical terms, even extensive testing is unlikely to reveal anything when there is no history or physical examination suggestive of an underlying pathological cause.^12^^,^^13^^,^^53^^,^^54^^,^^55^

As a routine, we recommend serum assaying of the following hormones: testosterone, free (bioavailable) testosterone, estradiol, hCG, LH, FSH, prolactin, T3, T4 and TSH. Testosterone, free (bioavailable) testosterone and LH should be measured in the morning, since they have a circadian rhythm with the highest values in the early hours.

### 3. Classifying into severity grades

GM shows a gradation of clinical types that range from simple areolar protrusion to breasts with a female appearance. The main clinical features characterizing GM are breast swelling, increased areolar diameter, presence of an anomalous inframammary fold, glandular ptosis and skin redundancy.

There are several morphological classifications for GM.^53^^,^^54^^,^^55^^,^^56^^,^^57^ For practical purposes, we have adopted the clinical classification of Cordova and Moschella,^56^ with some modifications, since this is simple and stringent. This classification takes into account the different relationships between the structural components of the breast, in particular the inframammary fold and nipple-areola complex (NAC), which is the watershed between mild forms and serious forms.

**Figure 2.**
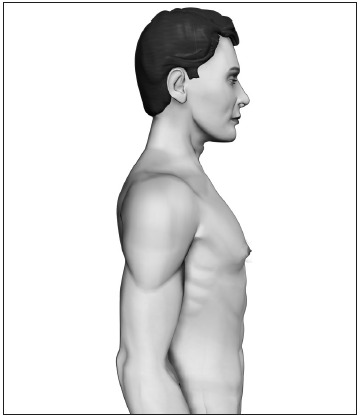
Grade I - Increased diameter and slight protrusion limited to the areolar region.

**Figure 3.**
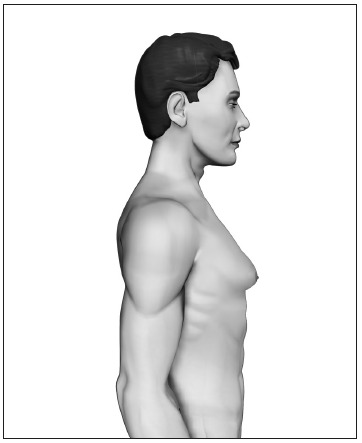
Grade II - Moderate hypertrophy of the breast with the nipple-areolar complex above the inframammary fold.

**Figure 4.**
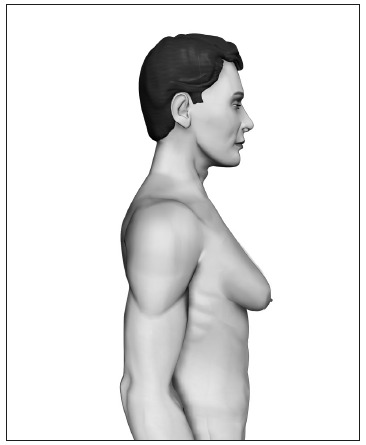
Grade III - Major hypertrophy of the breast with glandular ptosis and the nipple-areolar complex situated at the same height as or as much as 1 cm below the inframammary fold.

**Figure 5.**
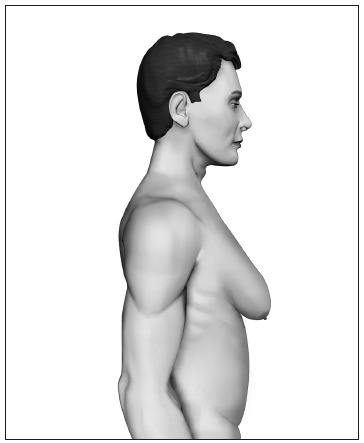
Grade IV - Major breast hypertrophy with skin redundancy, severe ptosis and the nipple-areolar complex positioned more than 1 cm below the inframammary fold.

Based upon this classification, in a standing position we classify all types of GM into four grades of increasing severity from I to IV, as follows (Figures 2, 3, 4 and 5):

- Grade I: Increased diameter and slight protrusion limited to the areolar region;
- Grade II: Moderate hypertrophy of all the structural components of the breast, with the NAC above the inframammary fold;
- Grade III: Major breast hypertrophy, glandular ptosis and the NAC at the same height as or as much as 1 cm below the inframammary fold;
- Grade IV: Major breast hypertrophy, with skin redundancy, severe ptosis and the NAC positioned more than 1 cm below the inframammary fold.

## TREATMENT

### General measures

If the GM is slight, without noteworthy psychological repercussion and the appropriate work-up does not reveal any underlying disease and only shows weight loss, reassurance and periodic follow-up visits (every 3-6 months) are recommended. Reassurance is widely regarded as the safest and most reasonable form of treatment, given that the condition is usually self-limiting and asymptomatic.^58^

For mild enlargements in pubertal boys, the simplest therapeutic approach is verbal explanation and reassurance regarding the signs and symptoms. Reassurance should be based on full and authoritative explanations about the common transient hormonal imbalance at this age, the natural involution of the condition and the absence of later sexual and fertility effects.^3^^-^^13^

Although fibroglandular overgrowth may also be present, this is typically overwhelmed in obese boys by the surrounding fat accumulation. For this reason and, equally as important, for other developmental and psychological reasons, a weight loss program (diet and physical exercises) should be the first-line treatment.

If possible, any causative medications or anabolic agents should be immediately withdrawn. Ceasing to use the offending agent may result in regression of GM.

If GM either persists or becomes more severe and is associated with pain, psychological distress or embarrassment caused by avoidance of activities in which the chest is exposed, pharmacological and surgical therapeutic options should be considered, especially when the patient wishes to pursue treatment.^17^

### Pharmacotherapy

With the assumption that there is a high possibility that the GM may spontaneously regress, the decision on when to treat is often difficult.^33^^-^^53^ However, medical treatment is likely to be beneficial if implemented during the early proliferative phase, before the glandular structure has been replaced by stromal hyalinization and fibrosis. Androgens, antiestrogens and aromatase inhibitors have been tested for GM treatment with some success.

Danazol is an antigonadotropic drug with a weak androgen effect that acts to counterbalance the stimulatory effects of estrogens. A daily dose of 200-600 mg may provide effective control over the symptoms.^58^^,^^59^ The side effects are acceptable. Dihydrotestosterone heptanoate, which does not undergo peripheral aromatization, was used in one study for a small number of pubertal cases with excellent results.^60^

Selective estrogen receptor modulators, such as tamoxifen and raloxifene are fairly safe and should be beneficial. The largest series reported is that of Alagaratnam,^61^ who treated 61 cases of idiopathic GM with tamoxifen (40 mg daily for 2-4 months) and achieved complete regression in 80% of the cases.^62^^,^^63^^,^^64^^,^^65^ Other prospective case series have reported success with tamoxifen at a dosage of 20 mg daily.

Two studies have compared tamoxifen with other substances. Ting et al.^64^ reported complete resolution of GM in 18 patients out of 23 treated with tamoxifen (78.2%), whereas the same regression was found in only eight out of 20 (40%) with danazol.^64^ Lawrence et al.^65^ retrospectively analyzed 38 adolescents with GM and found that some improvement was seen in 86% of the patients receiving tamoxifen and in 91% receiving raloxifene. Nevertheless, a greater proportion showed a significant decrease (> 50%) with raloxifene (86%) rather than with tamoxifen (41%).

Three studies have investigated the effectiveness of anastrozole, which is a potent aromatase inhibitor used for treating pubertal GM. While two observational studies with small samples have provided encouragement for the use of anastrozole,^66^^,^^67^ a well-designed randomized controlled trial on 80 pubertal boys found that anastrozole was not significantly more effective than placebo for reducing the breast volume calculated from ultrasonography measurements (38.5% versus 31.4%; P = 0.47).^68^

Tamoxifen was considered superior to anastrozole for prevention of bicalutamide-induced GM in men with prostate cancer.^69^ In a randomized placebo-controlled study, patients receiving tamoxifen presented a significantly reduced risk of GM after three months of treatment (20 mg daily), compared with those receiving placebo (risk relative 0.35; 95% confidence interval: 0.13-0.83), whereas the risk for patients treated with anastrozole was not significantly different from the placebo group.^70^

So far, no pharmacological agents for treating GM have been approved in the United States by the Food and Drug Administration. Given the small sample sizes and/or inadequacy of the methodology of published papers, there is no consensus regarding the drug of choice or the optimal duration of treatment. Overall, the use of drugs for GM treatment is only supported by very low quality of evidence, and the uncertainty about the balance between their benefits and potential harm should be highlighted to candidate patients.^17^ Nonetheless, in situations in which after discussing treatment options, scientific evidence, resources and the patient’s preference a medication is to be prescribed, we prefer tamoxifen.

### Surgical correction

GM of long duration is unlikely to regress spontaneously and may often progress to dense fibrosis and hyalinization. So far, for long-standing symptomatic GM, medical therapy is less likely to be effective because the stroma is mostly fibrotic. Traditionally, surgery has been the mainstay of therapy in such cases, as well as for men for whom medical therapy fails, is not tolerated or is declined, or for whom tissue removal is preferable for cosmetic reasons.^14^ Even during adolescence, surgery may be the preferred option. In some cases, denial of cosmetic correction or procrastination in providing this may create an additional unnecessary burden on an already overloaded psyche.^71^

The aims of surgical treatment of GM are to restore normal chest contours, eliminate the inframammary fold, correct the NAC position, remove redundant skin, create symmetry between the two halves of the chest and minimize scarring.^53^

There are many surgical techniques and treatment protocols for correcting GM in the literature.^54^^-^^57^^,^^69^^,^^73^^-^^78^ In most cases, considering that fibrous and fatty tissues need to be removed, the best results are achieved by combining liposuction and mammary adenectomy.^56^^-^^71^^,^^79^

The presence of cutaneous ptosis and the amount of excessive skin are decisive in guiding the choice between surgical treatment methods. In our opinion liposuction alone should not be used and is limited only to cases of pure pseudogynecomastia. Surgical treatment of gynecomastia requires an individualized approach.^80^

### Grade I

**Figure 6.**
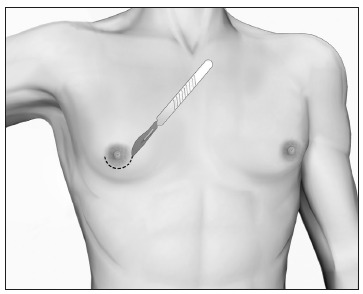
Mammary adenectomy by means of periareolar incision for gynecomastia grade I.

In grade I, the enlargement is caused solely by glandular proliferation without adipose accumulation. Surgical correction involves mammary adenectomy performed by a semicircular inferior periareolar incision. Liposuction is not required (Figure 6).

### Grade II

**Figure 7.**
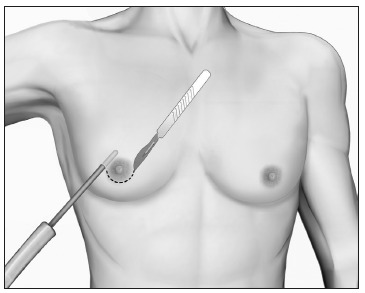
Liposuction followed by mammary adenectomy for gynecomastia grade II.

Grade II GM is characterized by excessive glandular tissue and local adiposity, with the NAC above the inframammary fold. In these cases, liposuction and surgical excision must be combined in the same operation (Figure 7). We recommend that the procedure should begin with vacuum lipoplasty and should be followed by mammary adenectomy by means of a classical periareolar incision. It is worth noting that other incisions may also allow good results, such as intra-areolar, pull-through or endoscopic procedures.^56^^,^^81^^,^^82^^,^^83^^,^^84^^,^^85^ The dissection flaps must be thin (0.5 cm), in order to avoid recurrences. Mammary adenectomy without liposuction leads to unsatisfactory outcomes, with an uneven surface or asymmetry.

### Grade III

In grade III, the operation begins with liposuction and is followed by glandular excision with periareolar removal of the tissue. It is necessary to detach the excess skin to obtain a good chest silhouette.

**Figure 8.**
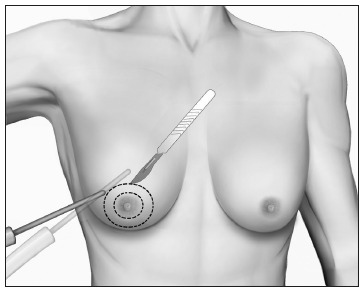
Liposuction followed by double-circle incision mammary adenectomy for gynecomastia grade III.

The surgical planning is undertaken bearing in mind the necessity to preclude the stigma of big scars. For the final appearance, it is always preferable for the scars to be restricted to the periareolar area. As a routine, a double-circle incision is performed over the skin, thereby making it possible to remove the epithelial tissue from an annular portion of skin that is as large as needed for each case (Figure 8). Because the epidermis has been removed, round-black suturing is done, which gives rise to a circumferential periareolar scar.^56^^,^^86^

### Grade IV

The hallmarks of grade IV GM are severe ptosis and a large amount of redundant skin. One of the techniques for reduction mastoplasty is used to remove gland and skin and flatten the chest outline.^56^^,^^57^^,^^85^

**Figure 9.**
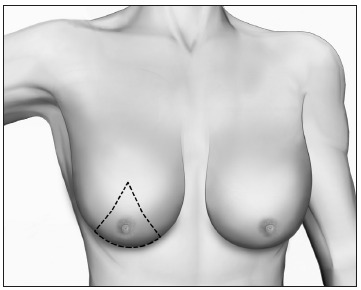
Inverted T-shaped mammary resection with nipple-areolar complex migration for gynecomastia grade IV.

Inverted T-shaped resection, with NAC migration using superior or superior-medial dermic pedicles, similarly to the procedure for mammoplasty in women, may be used. Other techniques such as those with horizontal or oblique incisions can also be used (Figure 9).

When patients strongly desire to have inconspicuous scars confined to the periareolar region, sequential double-circle skin incisions in a multi-step procedure are a valid option.

Since GM may be harboring neoplasia, histopathological analysis of the resected tissue is mandatory.

Closed suction drains are placed after the excisions and removed when the output over 24 hours has decreased to less than 30 ml. Up to 30% of the patients present postoperative complications, such as bleeding, hematoma and seroma formation.^87^ Large wound areas predispose towards these complications, especially in obese patients.

To facilitate tissue accommodation and achieve an even chest surface, we advise patients to wear elastic compressive garments for a period of 1-2 months. Hypoesthesia of the nipple is very common and is transient. The cosmetic results and patient satisfaction after surgery are high.

## CONCLUSIONS

GM is a common condition that may be attributable to an estrogen/androgen imbalance caused by several etiological factors. After confirming the diagnosis, searching for a specific cause and classifying the case according to severity grade, the therapy for GM should be personalized. Lifestyle guidance, reassurance, medical treatment and surgical correction are valid tailored therapeutic options.
